# Artificial Intelligence for Breast MRI Lesion Classification: A Targeted Evidence Synthesis and Meta-Analysis of Discriminative Performance and Heterogeneity

**DOI:** 10.3390/diagnostics16152316

**Published:** 2026-07-23

**Authors:** Romuald Ferre, Thad Benefield, Cherie M. Kuzmiak

**Affiliations:** 1 Division of Breast Imaging, Department of Radiology, UNC School of Medicine, Chapel Hill, NC 27599, USA; 2 Carolina Mammography Registry, University of North Carolina at Chapel Hill, Chapel Hill, NC 27599, USA; thad_benefield@med.unc.edu

**Keywords:** breast MRI, artificial intelligence, deep learning, lesion classification, diagnostic accuracy, meta-analysis, AUC

## Abstract

**Background/Objectives:** The paper aimed to synthesize the diagnostic performance of artificial intelligence (AI) methods for classifying breast lesions on contrast-enhanced breast MRI and to estimate a pooled area under the receiver operating characteristic curve (AUC). **Methods:** This targeted evidence synthesis and meta-analysis was informed by PRISMA 2020 reporting principles where applicable. Eligible studies were drawn from an investigator-supplied corpus of 12 primary manuscripts and assessed against predefined criteria. MEDLINE/PubMed, Embase, and Web of Science were consulted through October 2025 to contextualize the literature and verify bibliographic and study details; additional database records were not screened for eligibility. We included studies applying machine learning or deep learning to contrast-enhanced breast MRI for benign-versus-malignant lesion classification and reporting an AUC on an independent test set, external validation set, or patient-wise cross-validation. AUCs were pooled on the logit scale using an inverse-variance DerSimonian–Laird random-effects model, and heterogeneity was quantified using I^2^. **Results:** Nine studies met the criteria for quantitative synthesis (evaluation-set sizes, 60–3936). The pooled random-effects AUC was 0.898 (95% CI, 0.875–0.918), with substantial heterogeneity (I^2^ = 88.1%) and a 95% prediction interval of 0.824–0.943, indicating that performance may vary meaningfully across settings. **Conclusions:** AI models showed promising discriminative performance within this targeted corpus, but substantial heterogeneity, differences in unit of analysis, approximated variance estimates, and limited external institutional validation temper confidence in generalizability. The pooled AUC should be interpreted descriptively, and future studies should prioritize rigorous multi-institutional external validation, transparent reporting, and prospective reader- or workflow-impact evaluation before routine clinical deployment.

## 1. Introduction

Breast magnetic resonance imaging (MRI) is a highly sensitive modality for breast cancer detection and is increasingly used for screening of women at elevated risk, problem-solving, and preoperative staging [1]. Large population-based and high-risk screening trials have demonstrated the value of MRI in selected cohorts, including supplemental screening in women with extremely dense breast tissue [2]. Despite its sensitivity, breast MRI is resource-intensive: acquisition and interpretation are time-consuming, protocols are multiparametric, and interpretation requires integration of morphologic and kinetic features across sequences. Abbreviated breast MRI protocols leveraging early post-contrast subtraction images and maximum intensity projections (MIPs) have been proposed to mitigate scan and reading time, but adoption remains variable [3,4]. These practical barriers limit the scalability of breast MRI and motivate the development of tools that can improve workflow efficiency and diagnostic consistency.

Artificial intelligence (AI) has been investigated across multiple breast MRI use cases, including examination triage and lesion detection, lesion classification, tumor characterization, prediction of treatment response, risk assessment, and image quality improvement [5,6]. Deep-learning triage has been explored to exclude negative examinations and reduce radiologist workload [7,8,9,10]. A recent comprehensive review by Lo Gullo and colleagues summarized the rapidly expanding literature and highlighted substantial variability in datasets, imaging inputs (2D maximum intensity projections versus 3D volumes; single sequence versus multiparametric MRI), model architectures, and validation strategies [11]. Publicly available breast MRI datasets remain limited compared with mammography and ultrasound, which may hinder external validation and reproducibility [7].

Among these use cases, benign-versus-malignant lesion classification is particularly relevant because it can directly influence clinical decision making, including whether to recommend biopsy or short-interval follow-up. Individual studies often report high AUCs, but direct comparison is challenging due to heterogeneous cohorts (mass lesions versus non-mass enhancement), MRI protocols, and evaluation designs. Meta-analytic pooling of AUC provides a quantitative summary of discriminative performance and a framework to describe between-study variability.

The objective of this targeted evidence synthesis was to summarize the performance of AI-based models for breast MRI lesion classification within the investigator-supplied corpus and to estimate a pooled AUC using random-effects meta-analysis. We also summarized key study characteristics and examined factors likely to contribute to heterogeneity and barriers to clinical translation.

## 2. Methods

### 2.1. Reporting Guideline, Ethics, Protocol Registration, and Funding

This targeted evidence synthesis and meta-analysis was informed by PRISMA 2020 reporting principles where applicable. Because study identification relied on an investigator-supplied corpus rather than a comprehensive search-and-screening workflow, PRISMA items concerning complete search strategies and certainty-of-evidence assessment were applied only where relevant and qualified in the completed checklist [12].

**Figure 1:**
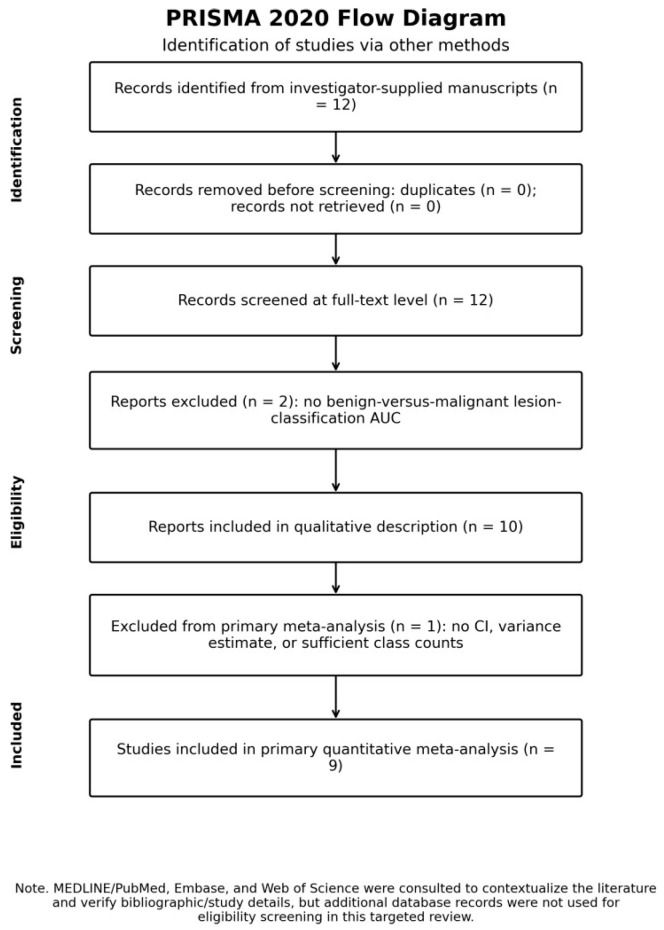
PRISMA-informed flow diagram for study identification, screening, eligibility, and inclusion. The selection process was based on a targeted investigator-supplied corpus of 12 primary manuscripts. MEDLINE/PubMed, Embase, and Web of Science were consulted to contextualize the literature and verify bibliographic and study details, but additional database records were not screened for eligibility.

A completed PRISMA 2020 checklist is provided as Supplementary File S1, and the PRISMA flow diagram is presented in Figure 1. The synthesis protocol was not registered, and no formal protocol was prepared. Because the study synthesized published, deidentified data, institutional review board or ethics committee approval was not required. No specific funding or external support was received.

### 2.2. Identification of Studies, Information Sources, and Selection Process

Eligible reports were drawn from a targeted investigator-supplied corpus of 12 primary manuscripts on AI-based breast MRI lesion classification. MEDLINE/PubMed, Embase, and Web of Science were consulted through October 2025 to contextualize the breast MRI AI literature and verify bibliographic and study characteristics when needed; these searches were not used to identify additional records for eligibility screening. No automation tools were used for screening or study selection.

All 12 reports were screened at the full-text level against the prespecified eligibility criteria. Screening decisions and reasons for exclusion were recorded and are summarized in the PRISMA flow diagram (Figure 1).

R.F. performed full-text screening, and C.M.K. verified eligibility decisions. Disagreements or uncertain decisions were resolved by consensus. Because the corpus was investigator-supplied, selection bias and incomplete capture of the available literature cannot be excluded [13,14,15].

### 2.3. Eligibility Criteria

We included studies meeting all of the following criteria:

- Population: patients undergoing contrast-enhanced breast MRI with lesions evaluated against histopathology or an accepted reference standard.
- Index test: AI-based model (machine learning or deep learning) applied to breast MRI inputs.
- Target condition: malignant breast lesion (binary classification, benign vs malignant).
- Outcome: AUC reported for benign-versus-malignant lesion classification evaluated on a held-out test set, external validation set, or patient-wise cross-validation.

We excluded studies if the primary objective was not benign-versus-malignant lesion classification (e.g., super-resolution or other image reconstruction tasks without diagnostic classification, molecular subtype prediction, or purely methodological work without a reported AUC for benign-versus-malignant discrimination).

### 2.4. Data Extraction and Handling of Multiple Results

Data were extracted at the study level and verified by the author team. Extracted items included first author, publication year, imaging inputs and sequences, model class or architecture when available, evaluation design (cross-validation, internal hold-out testing, or external validation), sample size, and the reported AUC for benign-versus-malignant lesion classification. When multiple eligible AUCs were available, priority was given to the author-designated primary model and the most independent validation setting (external validation, independent hold-out testing, then patient-wise cross-validation). Discrepancies or unclear items were resolved by consensus using the source reports.

### 2.5. Outcome Measures

The primary outcome was AUC. When a 95% confidence interval (CI) was reported, the standard error (SE) was estimated as (upper CI − lower CI)/(2 × 1.96). When uncertainty was not reported but malignant and nonmalignant class counts were available, the SE was approximated with the Hanley–McNeil variance estimator. For meta-analysis, SEs were transformed to the logit(AUC) scale using the delta method. These approximations assume independent observations and may underestimate uncertainty when multiple lesions per patient contribute to the evaluation set, class imbalance is present, or the effective sample size differs from the reported lesion or image count.

Secondary diagnostic-performance and clinical-utility outcomes were extracted when reported, including sensitivity, specificity, accuracy, positive and negative predictive values, calibration, reader-comparison results, biopsy recommendations, false-positive findings, recall rate, and workflow-impact measures. These outcomes were not pooled because reporting was inconsistent and often depended on study-specific thresholds.

### 2.6. Statistical Analysis

AUCs were pooled by inverse-variance weighting under fixed-effect and random-effects models. Because AUC is bounded between 0 and 1, analyses were performed on the logit(AUC) scale and back-transformed for presentation. Standard errors were converted using the delta method: SE_logit = SE_AUC/[AUC × (1 − AUC)]. The primary random-effects model used the DerSimonian–Laird estimator for between-study variance (τ^2^) [16]. Heterogeneity was assessed with Cochran’s Q and I^2^ [17], and a 95% prediction interval was calculated on the logit scale and back-transformed [18].

AUC was selected as the primary summary measure because it was the diagnostic-performance metric most consistently reported across the included studies. Hierarchical bivariate or hierarchical summary receiver operating characteristic meta-analysis was not performed because most reports did not provide threshold-specific sensitivity and specificity or 2 × 2 contingency-table data at comparable operating points. The pooled AUC should therefore be interpreted as a descriptive summary of discrimination within the targeted corpus rather than as a threshold-specific estimate of diagnostic accuracy.

DerSimonian–Laird was retained as the primary estimator to preserve consistency with the original analysis and because it remains widely used. However, with only nine studies and substantial heterogeneity, its normal-approximation confidence interval may be too narrow. Alternative approaches, including restricted maximum likelihood or Paule–Mandel estimation with Hartung–Knapp–Sidik–Jonkman adjustment, can provide more conservative uncertainty estimates in small, heterogeneous meta-analyses [19]. Because several within-study variances were approximated and the subset with directly reported uncertainty was too small for stable reanalysis, no formal alternative-estimator sensitivity model was presented. Interpretation therefore emphasized the prediction interval, heterogeneity, and the range of study-specific AUCs rather than the pooled confidence interval alone.

Formal meta-regression was not performed because only nine studies contributed to the quantitative synthesis, leaving insufficient degrees of freedom for reliable covariate modeling. Several clinically relevant strata were also sparse or non-overlapping. Potential drivers of heterogeneity were therefore summarized descriptively, including validation strategy, unit of analysis, MRI input representation, and external validation status.

Analyses were performed in Python 3.13 using NumPy 2.1.0, pandas 2.2.3, SciPy 1.14.1, and Matplotlib 3.9.2 for variance calculations, meta-analysis, heterogeneity assessment, prediction-interval calculation, and figure generation.

### 2.7. Reporting Quality and Risk of Bias Considerations

Risk of bias and applicability were assessed using the QUADAS-2 domains of patient selection, index test, reference standard, and flow and timing, together with AI-specific reporting considerations: patient-wise splitting, leakage prevention, handling of multiple lesions per patient, preprocessing before data partitioning, external validation, reproducibility, and code or trained-model availability. Assessments were made at the study level by the author team and resolved by consensus. Judgments were based on information explicitly reported in each paper; insufficiently reported items were classified as unclear. Key design and validation characteristics relevant to bias and generalizability are summarized in Table 1 [20,21].

### 2.8. Reporting Bias and Certainty Considerations

Because fewer than ten studies contributed to the primary quantitative synthesis, formal small-study-effect or publication-bias testing was not performed. Certainty in the body of evidence was not formally graded because the synthesis included retrospective diagnostic AI studies with heterogeneous units of analysis, MRI input representations, and validation designs. Confidence in generalizability was instead discussed narratively using risk-of-bias, applicability, external validation, and heterogeneity considerations.

## 3. Results

### 3.1. Study Selection

The study-selection process is shown in Figure 1. Twelve investigator-supplied reports were screened at the full-text level. No duplicate records were identified before screening. Two reports were excluded because they did not report an AUC for benign-versus-malignant lesion classification. Ten reports remained eligible for qualitative description; nine provided sufficient data for the primary quantitative synthesis. One additional study reported a challenge-based AUC without a corresponding CI, variance estimate, or sufficient class counts and was retained for narrative description but excluded from the primary meta-analysis [22,23,24,25,26,27,28,29,30,31,32,33].

### 3.2. Eligibility Decisions and Quantitative Synthesis Set

Among the ten eligible publications, all reported at least one relevant benign-versus-malignant AUC for breast MRI lesion classification. The primary meta-analysis included the nine studies with a usable variance estimate or sufficient data to approximate one. Table 1 summarizes the quantitative dataset and the primary AUC selected for each study; Figure 1 shows the reason one eligible report was excluded from the primary quantitative synthesis.

### 3.3. Study Characteristics

Most studies employed convolutional neural networks (CNNs). Input representations ranged from 2D maximum intensity projections (MIPs) derived from DCE-MRI to multiparametric MRI combinations and full 3D volumetric inputs. Evaluation-set sizes varied from 60 to 3936, reflecting differences in cohort selection and validation design (Table 1 and Table 2).

Important design differences included 2D MIP-based DCE-MRI inputs, multiparametric MRI inputs, and 3D CE-MRI volumes; lesion-level, case-level, non-mass-enhancement-lesion-level, and breast-level analyses; and patient-wise cross-validation, internal hold-out testing, scanner-based testing, and external institutional validation. These differences are summarized in Table 2 and were considered major sources of heterogeneity.

**Table 2:** Key design, validation, and applicability characteristics of studies included in the primary quantitative meta-analysis.

Study	Year	Design/Cohort	Center Status	Unit of Analysis	Validation Strategy	External Validation	Reference Standard
Antropova et al. [22]	2018	Retrospective; 2006–2016	Single center; University of Chicago	Lesion ROIs	5-fold cross-validation	No	Pathology
Dalmiş et al. [23]	2019	Retrospective; consecutive patients, 2011–2016	Single clinical center	Lesions	Patient-wise 5-fold cross-validation	No	Pathology + follow-up subset
Truhn et al. [24]	2019	Retrospective exams, 2011–2015; prospectively interpreted	Single academic breast center	Enhancing lesions	Patient-wise nested cross-validation	No	Histopathology or follow-up
Adachi et al. [25]	2020	Retrospective; 2014–2018; consent waived	Single hospital	Cases/lesions	Random train/validation/test split	No	Pathology or >1-year follow-up
Fujioka et al. [26]	2021	Retrospective; 2014–2018	Single hospital	Cases (MIP images)	Independent internal test set	No	Pathology or >1-year follow-up
Hu et al. [27]	2021	Retrospective	Single center; University of Chicago	Lesions	Temporal split + independent internal test set	No	Histopathology
Wang et al. [28]	2022	Retrospective; 2015–2020	Single center; two scanners	NME lesions	Random split + scanner-based test set	Partial; scanner-based only	Histopathology or follow-up
Zhu et al. [29]	2022	Retrospective multicenter	Multicenter + external institution	Lesions	Internal split + external institutional test set	Yes	Histopathology
Witowski et al. [30]	2022	Retrospective; 2008–2020	NYU Langone + external sites (US/Poland)	Breast-level labels	Internal patient-level split + external datasets	Yes	Pathology matched within 120 days

### 3.4. Quantitative Synthesis

**Figure 2:**
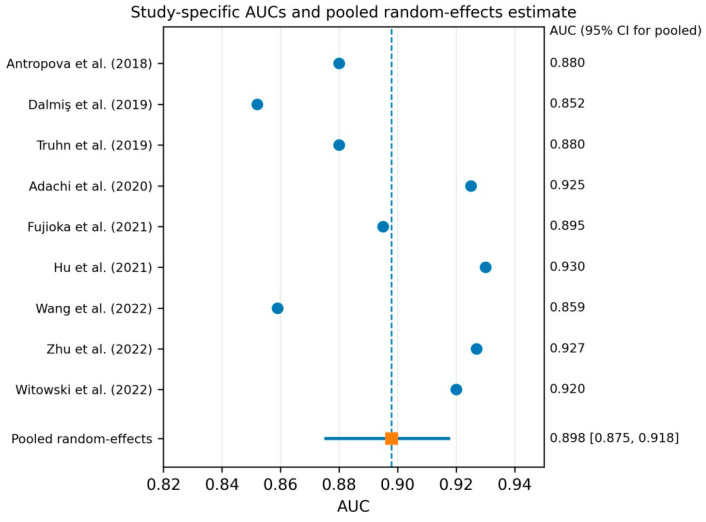
Study-specific AUC point estimates and the pooled DerSimonian–Laird random-effects AUC for AI-based breast MRI lesion classification [22,23,24,25,26,27,28,29,30]. Study-level confidence intervals are not displayed because uncertainty measures were not reported consistently; the pooled estimate is shown with its 95% confidence interval. Given the substantial heterogeneity and differences in validation strategy and unit of analysis, the pooled AUC should be interpreted descriptively.

Across the nine included studies, the DerSimonian–Laird random-effects AUC was 0.898 (95% CI, 0.875–0.918). Heterogeneity was substantial (Q = 67.38; I^2^ = 88.1%; τ^2^ = 0.089 on the logit scale), and the 95% prediction interval was 0.824–0.943. Given the limited number of studies, approximated variance estimates for some reports, and differences in unit of analysis and validation strategy, the pooled AUC should be interpreted as a descriptive summary of the targeted corpus rather than as a transferable clinical-performance estimate (Figure 2).

Formal meta-regression was not performed because the number of studies was small. Descriptive stratification showed overlapping AUC ranges across categories: 0.859–0.930 for 2D MIP-based approaches, 0.852–0.927 for multiparametric MRI approaches, and 0.920 for the single 3D volumetric study. Studies with external institutional validation reported AUCs of 0.920–0.927; those without external validation or with scanner-based testing reported 0.852–0.930. Lesion- or non-mass-enhancement-lesion-level studies reported 0.852–0.930, case- or MIP-image-level studies 0.895–0.925, and the single breast-level study 0.920. These overlapping ranges suggest that heterogeneity is multifactorial and cannot be attributed to a single characteristic.

A sensitivity analysis excluding studies with approximated variance estimates was considered but not performed because too few studies would have remained for a stable pooled model. The influence of variance approximation was therefore addressed narratively and is emphasized as a limitation.

Although AUC was the most consistently reported metric, other diagnostic-performance and clinical-utility outcomes were reported inconsistently. Sensitivity, specificity, accuracy, predictive values, calibration, reader-comparison results, biopsy recommendations, false-positive findings, recall rate, and workflow impact were not available in a sufficiently standardized format for quantitative pooling and were therefore summarized narratively.

The absence of consistent threshold-specific reporting limits the assessment of how AI-based lesion classification would affect biopsy decisions, follow-up recommendations, false-positive reduction, or radiologist workflow in clinical practice.

### 3.5. Risk of Bias and Applicability

QUADAS-2 judgments are summarized in Table 3. Flow and timing were the most frequent source of risk-of-bias concern, rated high or unclear in seven of nine studies, followed by the reference-standard domain in five studies and patient selection in four; index-test risk was unclear in one study and low in the remainder. Applicability concerns were less frequent: patient selection was rated high for Antropova et al., and Dalmiş et al., index-test applicability was high for Witowski et al., and reference-standard applicability was unclear for Fujioka et al. These patterns indicate that incomplete participant flow, timing, and reference-standard reporting were more prominent constraints than index-test conduct itself. Because unclear judgments often reflected incomplete reporting rather than confirmed flaws, the direction and magnitude of bias cannot be quantified, but confidence in the pooled estimate and its generalizability is reduced.

Most studies were retrospective and single-center, and several used cross-validation or internal test sets rather than fully independent external validation. Only Zhu et al. and Witowski et al. evaluated external institutional datasets; Wang et al. used a scanner-based test set that addresses technical domain shift but not broader institutional variation. Differences in lesion-, case-, and breast-level analysis further limit direct comparison of AUCs.

AI-specific sources of bias were also incompletely reported. Multiple lesions or images from the same patient may be correlated, and performance can be optimistic when patient-wise separation is not strictly maintained. Preprocessing leakage can arise when normalization, segmentation, augmentation, feature extraction, lesion selection, or hyperparameter tuning is informed by data outside the training partition. Limited reporting of code, model weights, and reproducibility procedures further restricts independent validation.

## 4. Discussion

This targeted evidence synthesis found promising discrimination for AI-based breast MRI lesion classification, with a DerSimonian–Laird random-effects AUC of 0.898 (95% CI, 0.875–0.918). However, heterogeneity was substantial (I^2^ = 88.1%), and the 95% prediction interval (0.824–0.943) indicates that performance in a new setting could vary materially. The pooled AUC should therefore be interpreted as a descriptive summary of the investigator-supplied corpus rather than as a transferable benchmark for clinical practice.

The heterogeneity is clinically and methodologically plausible. Studies differed in MRI inputs (2D MIPs, multiparametric combinations, and 3D volumes), lesion spectrum, reference standards, unit of analysis, validation strategy, and institutional setting. Lesion-level, case-level, and breast-level AUCs are not directly interchangeable, and retrospectively assembled lesion-enriched cohorts may produce higher discrimination than unselected screening or surveillance populations. Descriptive stratification did not identify a single factor that explained the variability.

Generalizability was further limited by the scarcity of genuine external institutional validation. Only Zhu et al. and Witowski et al. evaluated models on external institutional datasets; Wang et al. used a scanner-based test set that addresses technical domain shift but not the broader variation encountered across institutions. Performance may change with scanner vendor, field strength, acquisition protocol, contrast timing, patient population, lesion spectrum, and interpretation workflow.

The QUADAS-2 findings reinforce this caution. Flow and timing were high or unclear in seven studies, reference-standard risk was high or unclear in five, and patient-selection risk was high or unclear in four. These judgments suggest that cohort assembly, verification pathways, and incomplete reporting of participant flow may influence the reported AUCs. Applicability concerns were concentrated in a small number of studies, but the predominance of retrospective, lesion-enriched cohorts still limits translation to screening populations.

AI-specific methodological limitations also matter. Multiple lesions or images from the same patient may be correlated, and performance can be optimistic when patient-wise separation is not strictly maintained. Preprocessing leakage can arise when normalization, segmentation, augmentation, feature extraction, lesion selection, or hyperparameter tuning is informed by data outside the training partition. Limited reporting of code, model weights, and reproducibility procedures further restricts independent validation.

AUC summarizes discrimination across thresholds but does not establish a clinically useful operating point. Sensitivity, specificity, predictive values, calibration, biopsy recommendations, false-positive reduction, recall, and workflow outcomes were too inconsistently reported for pooling. Reader-comparison studies within the corpus suggest potential adjunctive value for AI [25,26], but prospective trials are needed to determine whether AI improves decisions or efficiency without compromising cancer detection.

This synthesis has several limitations. The 12-report evidence base was investigator-supplied rather than generated through a comprehensive search-and-screening workflow, so selective availability and incomplete literature capture cannot be excluded. The pooled analysis combined heterogeneous cohorts and units of analysis, and several within-study variances were approximated under assumptions that may not account for class imbalance or within-patient clustering. DerSimonian–Laird may produce overly narrow confidence intervals when the number of studies is small, and heterogeneity is substantial; accordingly, inference emphasized the prediction interval, heterogeneity, and study-specific range rather than the pooled confidence interval alone. A sensitivity analysis limited to studies with directly reported uncertainty was considered, but could not be performed reliably because too few studies would have remained. Publication bias was not formally assessed because fewer than ten studies were included.

Future work should prioritize multi-institutional external validation, patient-wise data partitioning, explicit handling of multiple lesions, transparent preprocessing, and reporting of calibration and clinically prespecified operating thresholds. Prospective reader- and workflow-impact studies, supported by standardized public datasets and reproducible benchmarking, are needed before routine clinical deployment [7,20,21].

## 5. Conclusions

AI methods for breast MRI lesion classification showed promising discrimination within this targeted evidence base. However, the pooled AUC is a descriptive estimate because the corpus was investigator-supplied, heterogeneity was substantial, units of analysis differed, several variances were approximated, and external institutional validation was limited. Current evidence supports further multi-institutional validation rather than routine clinical deployment. Future studies should use strict patient-wise data partitioning, report calibration and clinically relevant operating thresholds, and evaluate reader and workflow outcomes prospectively.

## Figures and Tables

**Table 1:** Studies included in the primary quantitative meta-analysis and primary AUC selected for pooling.

Study	Year	MRI Input Representation	Evaluation Sample Size, *n*	Unit of Analysis	Primary Validation Setting	Primary AUC
Antropova et al. [22]	2018	2D MIP (DCE-MRI)	*690*	Lesion ROIs	5-fold cross-validation	0.880
Dalmiş et al. [23]	2019	Multiparametric MRI (DCE + T2 + DWI)	*576*	Lesions	Patient-wise 5-fold cross-validation	0.852
Truhn et al. [24]	2019	Multiparametric MRI (DCE + T2 + DWI)	*1294*	Enhancing lesions	Patient-wise nested cross-validation	0.880
Adachi et al. [25]	2020	2D MIP (DCE-MRI)	*180*	Cases/lesions	Random train/validation/test split	0.925
Fujioka et al. [26]	2021	2D MIP (DCE-MRI)	*60*	Cases (MIP images)	Independent internal test set	0.895
Hu et al. [27]	2021	2D deep-feature MIP (DCE-MRI)	*1990*	Lesions	Temporal split + independent internal test set	0.930
Wang et al. [28]	2022	2D MIP (DCE-MRI), NME	*362*	NME lesions	Random split + scanner-based test set	0.859
Zhu et al. [29]	2022	Multiparametric MRI (DCE + DWI) + segmentation	*423*	Lesions	Internal split + external institutional test set	0.927
Witowski et al. [30]	2022	3D CE-MRI volumes	*3936*	Breast-level labels	Internal patient-level split + external datasets	0.920

Abbreviations: AUC = area under the receiver operating characteristic curve; CE-MRI = contrast-enhanced MRI; CV = cross-validation; DCE-MRI = dynamic contrast-enhanced MRI; DWI = diffusion-weighted imaging; MIP = maximum intensity projection; NME = non-mass enhancement; ROI = region of interest.

**Table 3:** QUADAS-2 assessment summary. Legend: Low = low risk or concern; High = high risk or concern; Unclear = insufficient information reported.

(**a**) Risk of Bias				
**Study**	**Patient Selection**	**Index Test**	**Reference Standard**	**Flow & Timing**
Antropova et al. (2018) [22]	High	Unclear	Low	Unclear
Dalmiş et al. (2019) [23]	High	Low	Unclear	High
Truhn et al. (2019) [24]	High	Low	Unclear	High
Adachi et al. (2020) [25]	Low	Low	Unclear	High
Fujioka et al. (2021) [26]	Low	Low	High	High
Hu et al. (2021) [27]	Low	Low	Low	Low
Wang et al. (2022) [28]	Low	Low	Unclear	High
Zhu et al. (2022) [29]	Unclear	Low	Low	Low
Witowski et al. (2022) [30]	Low	Low	Low	Unclear
(**b**) Applicability concerns				
**Study**	**Patient Selection**	**Index Test**	**Reference Standard**	
Antropova et al. (2018) [22]	High	Low	Low	
Dalmiş et al. (2019) [23]	High	Low	Low	
Truhn et al. (2019) [24]	Low	Low	Low	
Adachi et al. (2020) [25]	Low	Low	Low	
Fujioka et al. (2021) [26]	Low	Low	Unclear	
Hu et al. (2021) [27]	Low	Low	Low	
Wang et al. (2022) [28]	Low	Low	Low	
Zhu et al. (2022) [29]	Low	Low	Low	
Witowski et al. (2022) [30]	Low	High	Low	

## Data Availability

The extracted study-level data and analytic code are available from the corresponding author upon reasonable request.

## Supplementary Materials

The following supporting information can be downloaded at: https://www.mdpi.com/article/10.3390/diagnostics16152316/s1, File S1: Completed PRISMA 2020 checklist.
